# 

**DOI:** 10.1192/bjb.2022.26

**Published:** 2023-04

**Authors:** Rebecca Lawrence

**Affiliations:** is consultant psychiatrist with NHS Lothian, Edinburgh, UK. Email: rebecca.lawrence@nhslothian.scot.nhs.uk

**Figure:**
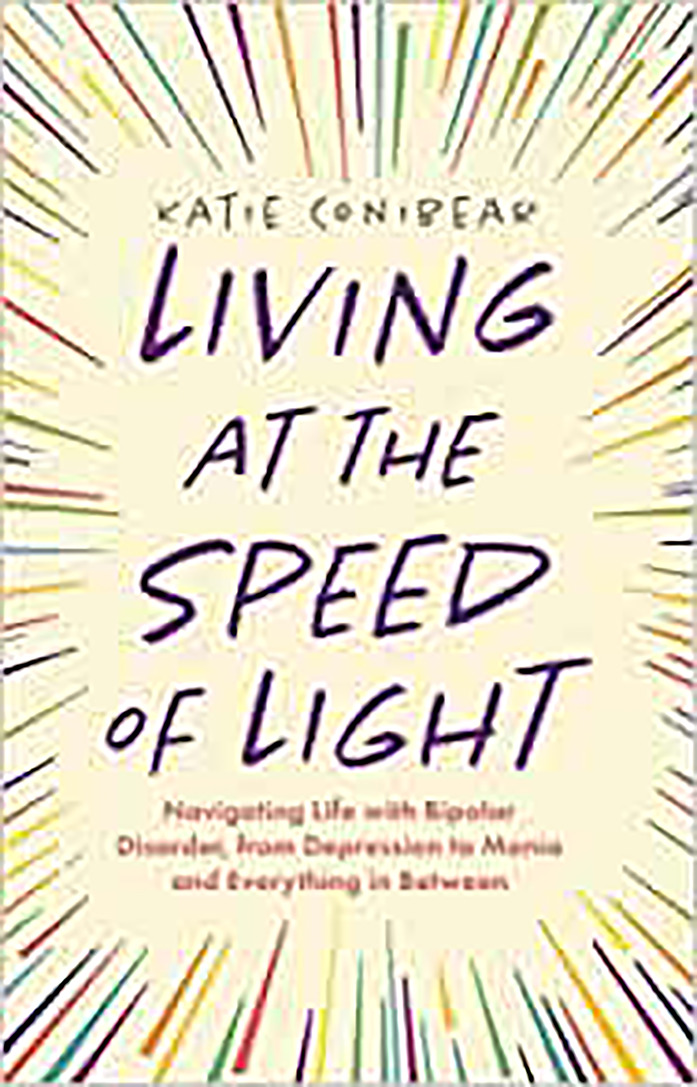

*Living at the Speed of Light*’, by Katie Conibear, is a friendly and accessible guide for people with bipolar disorder and their friends and families. It is written in a style that is easy to read, with plenty of bullet points and summaries, and provides a wealth of well-researched information. However, it is the author's descriptions of her own experiences, which are scattered throughout, that make it particularly compelling. It is not a textbook and it largely avoids topics like the Mental Health Act, complex medical therapies and the experience of in-patient treatment, in my view a wise decision. The chapters are well structured, and although the author touches on aspects such as aetiology and pharmacology, the focus is on practical and emotional aspects.

Getting a diagnosis can be challenging and it is important, as a psychiatrist, to appreciate the relief that the author felt on finally being given this label – which she described as a ‘release’ – after 12 years of symptoms. Her descriptions of both mania/hypomania and depression are excellent, and well-illustrated with her own examples. She gives helpful advice regarding recognising warning signs and triggers, and does not avoid the uncomfortable – her description of phoning for help when suicidal and being directed from pillar to post makes unpleasant reading.

The second half of the book is very informative regarding day-to-day management of life, work and other people. I was very struck by the author's criticism of the concept of being called ‘high functioning’, particularly with a disorder that is, by nature, variable; and also by her positive approach to managing stigma. She gives a lot of good advice about work and education, and is always careful to emphasise that different people will experience things differently. But the difficulties that she has experienced shine through – particularly in her heartfelt cry ‘You shouldn't have to “come out” with bipolar’.

The penultimate chapter deals with staying well, and I embrace her view that management is a better description than recovery. The last chapter stands on its own as an excellent resource for families and friends, a view endorsed by my husband, who has many years of living with someone with bipolar disorder.

In summary, I would strongly recommend this book to people of all ages with bipolar disorder and to anyone else who wants to better understand their experiences.

